# Isolated myeloid sarcoma with pericardial and pleural effusions as first manifestation: A case report

**DOI:** 10.1097/MD.0000000000031026

**Published:** 2022-10-21

**Authors:** Kunyi Deng, Wenpeng Ni, Lilian Li, Yanhui Chen, Li Wang, Wendong Ju

**Affiliations:** a Clinical Laboratory, Zhongshan Boai Hospital Affiliated to Southern Medical University, Zhongshan, Guangdong, China; b Department of Hematology, Zhongshan Boai Hospital Affiliated to Southern Medical University, Zhongshan, Guangdong, China.

**Keywords:** acute myeloid leukemia, cytomorphology, effusion, myeloid sarcoma

## Abstract

Myeloid sarcoma (MS) involves the proliferation of extramedullary blasts from 1 or more myeloid lineages, replacing the original tissue structures, and these neoplasias are called granulocytic sarcoma, chloroma, or extramedullary myeloid neoplasms. These tumors develop in lymphoid organs, bones, skin, soft tissues, various mucous membranes, organs, and the central nervous system. MS is rare in non-leukemic patients, while MS patient with effusion as the first manifestation is even rare.

**Patient concerns:**

We report the case of 44-year-old woman with abdominal pain, diarrhea, and vomiting.

**Diagnosis:**

Ultrasound examination and computed tomography of the chest revealed large pericardial effusions and bilateral pleural effusions. Cytomorphological examination of the pericardial and pleural effusion, flow cytometry, and immunohistochemical markers suggested myeloid tumor cells. However, concurrent peripheral blood and bone marrow examinations showed no evidence of acute myeloid leukemia. The patient was eventually diagnosed with isolated MS.

**Interventions and outcomes:**

After chemotherapy with pirarubicin + cytarabine and high-dose cytarabine + etoposide, the pericardial effusion and pleural effusion were absorbed, and the mediastinal mass significantly shrunk. One year after patient gave up treatment, acute myeloid leukemia (AML) was confirmed by bone marrow examinations.

**Conclusion:**

The early manifestations of the patient lacked specificity and were highly susceptible to misdiagnosis. Cytomorphology and flow cytology indicated important directions for the diagnosis of the disease in the early stage. Administration of chemotherapy regimen containing cytarabine could prolong disease-free survival and time before progress to AML.

## 1. Introduction

Myeloid sarcoma (MS) is an extramedullary tumor composed of various combinations of myeloblasts, myeloid precursors, and neutrophils that involves the proliferation of extramedullary blasts from 1 or more myeloid lineages to replace the original tissue structure. The tumor was first reported by Burn in 1811, and King called it “green tumor” in 1853. In 1966, Rappaport proposed the term “granulocytic sarcoma”, and it was not until 2002 that the World Health Organization announced the name “myeloid sarcoma”.^[1]^ Myeloid sarcomas can affect almost any part of the body. The most common sites are the skin, lymph nodes, gastrointestinal tract, bone, soft tissue, and testes. In less than 10% of cases, myeloid sarcomas arise in multiple anatomical sites.^[2]^ Many MS patients have either coexisting acute myeloid leukemia (AML) or a myeloproliferative or myelodysplastic disorder at the time of diagnosis, or these tumors appear at the first sign of relapse from one of these disorders. Here, we report a rare case of isolated MS with pericardial and pleural effusion as first manifestation.

## 2. Case presentation

The patient, a 44-year-old female, was admitted to the hospital for 2 days of cough and asthma, accompanied by abdominal pain and vomiting for 1 day, without chills, fever or other symptoms. Physical examination: orthopnea, hepatojugular reflux, enlargement of the left cardiac border, heart rate 103 beats/minute, weak and distant heart sound.

Laboratory tests: decreased red blood cell count and hemoglobin, decreased serum iron and ferritin, and increased soluble transferrin receptors. Liver enzymes, renal function and electrolytes were within normal limits. Ultrasonography and computed tomography (CT) scan of the chest showed large pericardial effusions, widened upper mediastinum with high density, moderate bilateral pleural effusions, and segmental atelectasis in the lower lobe of the right lung.

**Figure 1.**
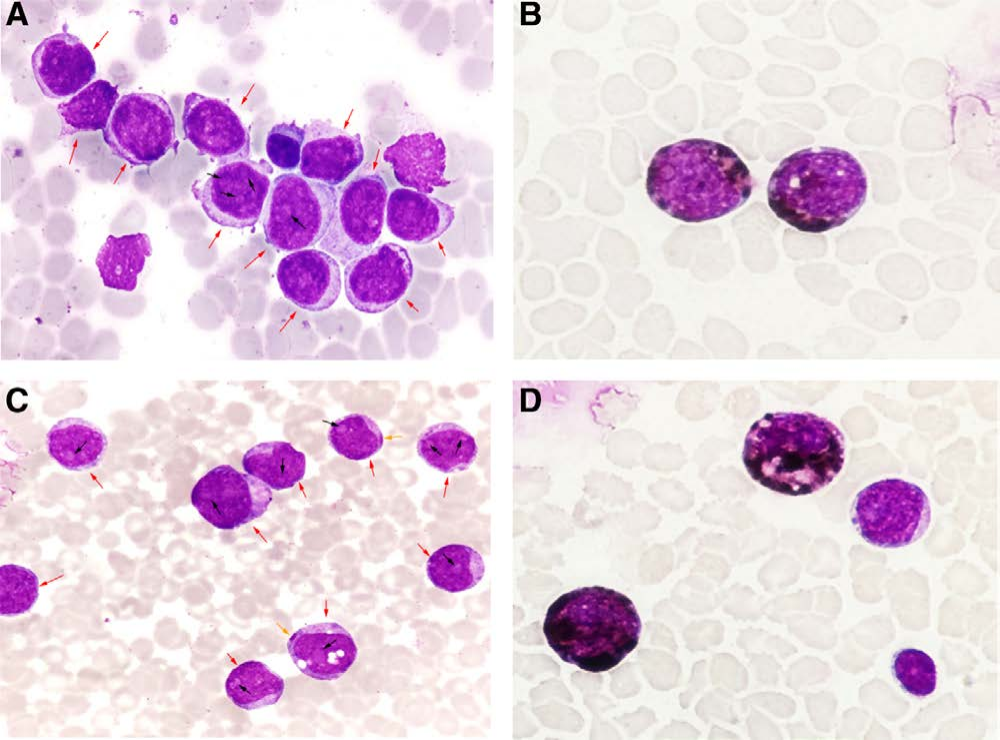
Cytomorphological examination of pericardial effusion (A) and pleural effusion (C), Wright staining, ×1000; POX staining of pericardial effusion (B) and pleural effusion (D), POX staining, ×1000. (The red arrows point to blast cells, the black arrows point to nucleoli, and the yellow arrows point to Auer rods.) POX = positive peroxidase.

**Figure 2.**
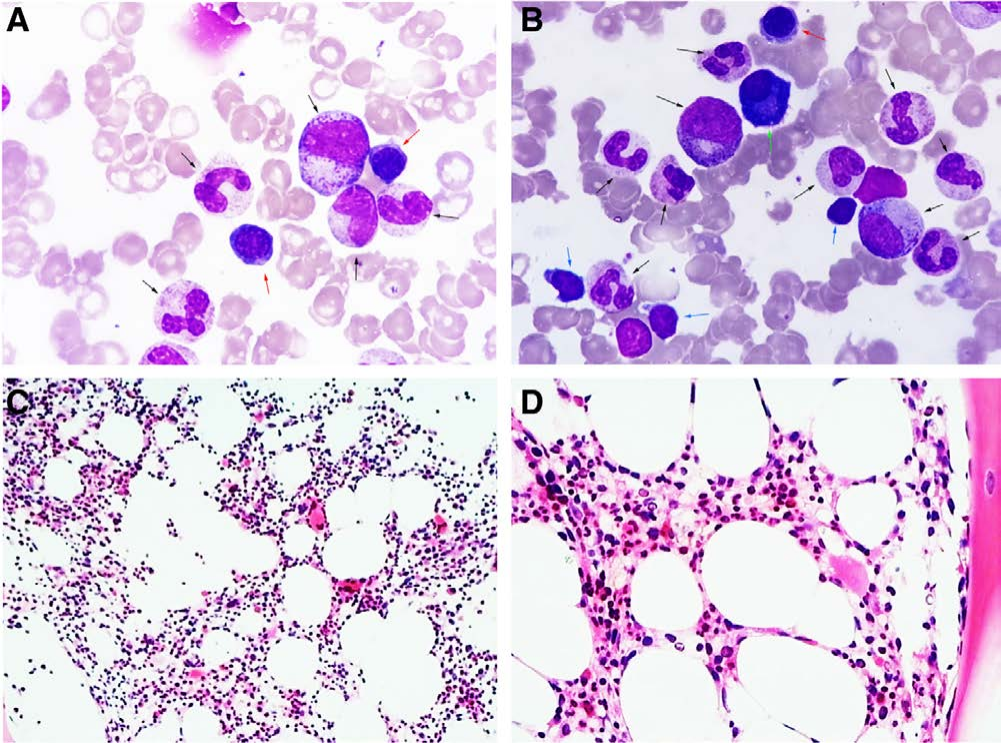
Images from the smears and biopsies of bone marrow. The images show that the bone marrow is normal without acute myeloblastic leukemia. Bone marrow smear, Wright and Giemsa stain, ×1000 (A, B); Bone marrow biopsy, Wright and Giemsa staining, ×200 (C), ×400 (D). (The red arrows point to erythrocytoblasts, the black arrows point to granulocytes, the blue arrows point to lymphocytes, and the green arrow point to plasma cell.)

**Figure 3.**
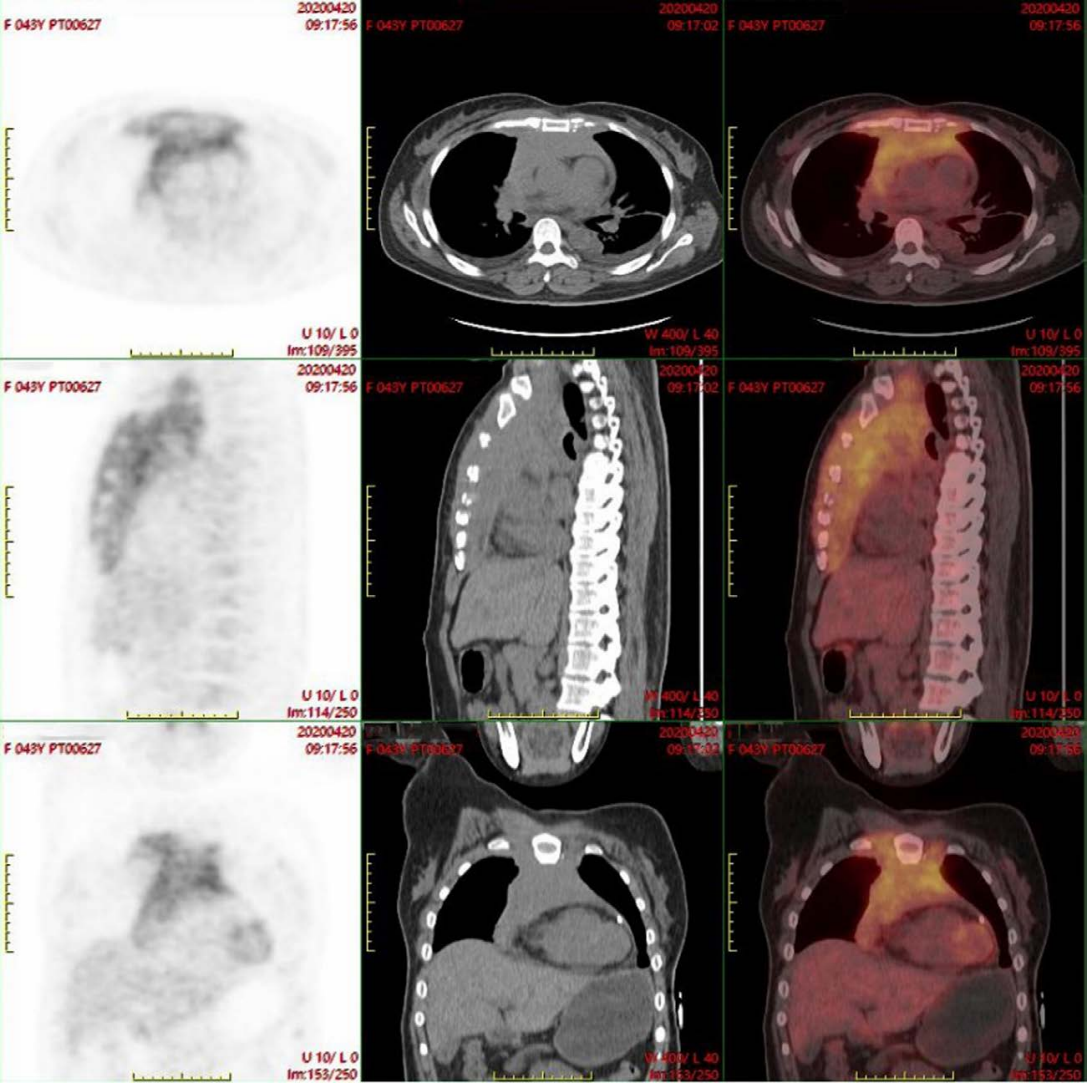
Images from the pectoral PET/CT. Irregular stripe-like softtissue thickening is seen in the anterior mediastinum, mostly located in the anterior superior mediastinum, with diffuse and heterogeneous increased fluorodeoxyglucose uptake, the size is about 8.4 cm × 3.2 cm × 16.3 cm, and the maximum standard uptake value is about 5.8. PET/CT = positron emission tomography/computed tomography.

**Figure 4.**
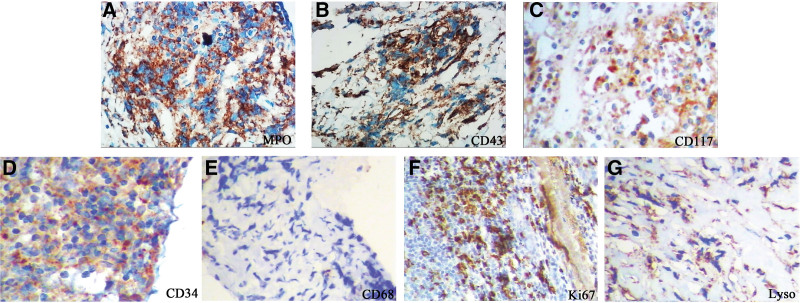
Immunohistochemical characteristics of mediastinal mass puncture specimens. (A) Staining for MPO; (B) Staining for CD43; (C) Staining for CD117; (D) Staining for CD34; (E) Staining for CD68; (F) Staining for Ki67; (G) Staining for Lyso. ×400 for all images. MPO = myeloperoxidase.

On the third day of admission, the patient developed pericardial tamponade and worsened dyspnea, and a pericardial cavity puncture and drainage was performed, and 100 mL of bloody turbid fluid was withdrawn. After the procedure, blood pressure and blood oxygen decreased, and cardiogenic shock was considered, and the patient was transferred to the intensive care unit for oxygenation, rehydration, and monitoring of vital signs. Cytomorphological examination of the pericardial effusion showed a large number of immature cells (about 70%), which had clear nucleoli ranging from 1 to 3, a high nucleoplasmic ratio, and about 46% were positive peroxidase (POX) (Fig. 1A, B). Flow cytometry showed that 38.9% of myeloblasts were positive for clusters of differentiation (CD)34, CD117, CD33, human leukocyte antigen-DR (HLA-DR), a small amount of cytoplasmic myeloperoxidase (MPO), and partially positive for CD64 and CD56. Five days later, the patient’s dyspnea improved, and bilateral thoracentesis and catheter drainage were performed to drain 500 mL of bloody fluid. Cytomorphological examination of the pleural effusion showed a diffuse distribution of a large number of mononuclear-like cells with large hyperchromatic nuclei, fine chromatin and sparse eosinophilic cytoplasm (Fig. 1C, D). About 66.9% of myeloid blasts were found in pleural effusion, and the immunophenotype was positive for CD34, CD117, CD33, CD64, HLA-DR, a small amount of cytoplasmic MPO, and partially positive for CD56 (Fig. S1; http://links.lww.com/MD/H835). No abnormalities were found in 2 cytomorphological examinations of bone marrow (Fig. 2A, B), and no myeloid blasts with abnormal immunophenotype were detected by flow cytometry (Fig. S2; http://links.lww.com/MD/H836). Decreased marrow iron assessed by Prussian blue staining, and bone marrow biopsy showed no abnormal localization of immature precursor cells and no collagen fibrosis in the bone marrow interstitium (Fig. 2C, D). Positron emission tomography/computed tomography (PET/CT) scan was performed on the 20th day of the patient’s admission, and the imaging results are shown in Figure 3. Mediastinal biopsy was performed on the 21st day, and the pathological report suggested that the patient was highly suspected of malignant tumor. Immunohistochemistry showed MPO, CD43, CD117, CD34, Lyso positive, CD68 negative, Ki-67 positive index 40% (Fig. 4).

**Figure 5.**
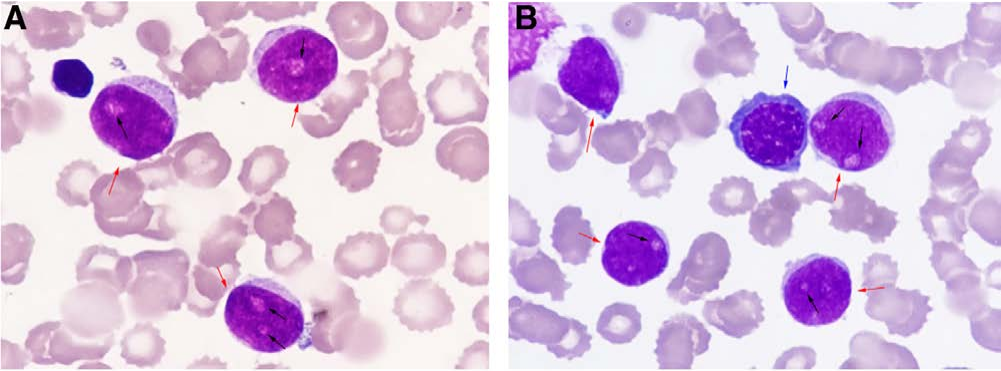
Images from the bone marrow smears. (The blue arrow points to erythrocytoblast, the black arrows point to nucleoli, and the red arrows point to blast cells, Wright and Giemsa staining, ×1000).

Based on the above data, the final diagnosis was isolated MS. The TA chemotherapy regimen (pirarubicin 60/50/30 mg/m^2^ qd on days 1–3 + cytarabine 150 mg/m^2^ qd on days 1–6) was used on day 23. After 1 course of treatment, CT scan of the chest showed that the tumor in the anterior mediastinum was significantly smaller than before, the pericardial effusions were significantly reduced, and the bilateral pleural effusions had been basically absorbed. Subsequently, TA regimen chemotherapy (pirarubicin 60/50/30 mg/m^2^ qd on days 1–3 + cytarabine 150 mg/m^2^ qd on days 1–6) was used again, and 37 days later, EA regimen (cytarabine 1 g/m^2^ q12h on days 1, 3, 5 + etoposide 100 mg/m^2^ qd on days 1, 3, 5) was used for one course of chemotherapy. After treatment, the patient had no symptoms such as cough and asthma, and his physical condition improved significantly. Unfortunately, the patient did not continue treatment for economic reasons. Follow-up: 1 year and 8 months after discharge, the patient developed right lower extremity swelling and pain for 1 week, coughing for 2 days, and blood vessel color ultrasound showed venous thrombosis in the right lower extremity. Complete blood count showed leukocyte was 26.3 × 10^9^/L, the hemoglobin was 92 g/L, the platelet was 43 × 10^9^/L, and the absolute value of monocyte was 15.25 × 10^9^/L. CT scan of the chest showed mass in the anterior mediastinum, moderate pleural effusion on both sides, especially on the left side, and pericardial effusion. Cytomorphological examination of bone marrow suggested AML (Fig. 5). Flow cytometry of bone marrow showed that CD34-positive cells accounted for 81.5% of the total nuclear cells, and their immunophenotype was positive for CD34, CD117, HLA-DR, and CD56, and partially positive for CD64 (Fig. S3; http://links.lww.com/MD/H837). AML-related gene mutation detection showed: TP53, PHF6 mutation were positive, other mutations were negative, fusion gene mutation were negative. Flow cytometry of pleural effusion showed about 87.8% of myeloid blasts, and their immunophenotypes were positive for CD34, CD117, CD33, and CD56, and partially positive for HLA-DR and CD11b. Considering progression to AML, the patient refused chemotherapy and signed off for discharge.

## 3. Discussion

The 2017 revision of the World Health Organization’s classification of myeloid neoplasms and acute leukemia separately classified MS as a special subtype of AML, and proposed that isolated MS could be equivalent to AML.^[3]^ It usually takes 5 to 12 months for primary MS to progress to AML.^[4]^ Because of the lack of hematological manifestations of myeloid leukemia, it is easy to be misdiagnosed as lymphoma, Ewing sarcoma, thymoma or other poorly differentiated cancers, the rate is 25% to 47%.^[5,6]^ It has been reported that there is no gender difference in patients with primary or isolated MS and that it is more common in young and middle-aged people,^[7]^ which is different from the increasing incidence of AML with age.

The diagnosis of isolated MS is based on histopathological and immunohistochemical examinations. From a cytomorphological point of view, the possibility of MS should be considered when the tumor cells resemble lymphoid tumor cells but cannot be classified into any type of lymphoid tumor. MPO is the marker with the highest sensitivity and specificity for IMS, with positive expression in 66% to 96% of patients, which can be distinguished from lymphoma. The positive expression of other markers such as lysozyme, CD43, CD117, and CD68 can help identify isolated MS.^[8]^ CD34 can be expressed in lymphoid and myeloid tumor cells,^[8]^ so it is believed that CD34 is not helpful for the diagnosis of MS. Serous cavity effusion can be the result of an isolated disease or systemic diseases such as metastases from malignant tumors like lung cancer, breast cancer, and lymphoma.^[9]^ Acute and chronic leukemias and myelodysplastic syndromes rarely involve sites such as the pleural cavity. This patient presented with serous cavity effusion combined with mediastinal mass as the first manifestation, and similar case reports of MS are rare.^[10]^

In this case, after chemotherapy with TA and EA, the pericardial effusion and pleural effusion were absorbed, and the mediastinal mass significantly shrunk. The study by Sahu et al^[11]^ showed that cytarabine may be an important drug for the treatment of MS. One year after stopping chemotherapy, this patient was diagnosed with AML, which is consistent with the results of previous studies.^[12]^ AML-related gene examination of the patient showed TP53 mutation and PHF6 mutation. The PHF mutation rate in AML was about 3%, indicating a poor prognosis. MS is a local manifestation of systemic disease, and early standardized chemotherapy is extremely important. Antic et al^[6]^ reported that patients who received regular systemic chemotherapy had a much higher disease-free survival rate than patients who only received local surgery or radiotherapy. The study of Lee et al^[12]^ showed that patients who received chemotherapy alone or chemotherapy combined with local therapy still had a higher risk of recurrence and progress to AML, while patients who received transplantation had a significant improvement in complete remission status. Most researchers believe that hematopoietic stem cell transplantation is more beneficial for MS patients to obtain a good prognosis.^[13,14]^ The early manifestations of our patient lacked specificity and was highly susceptible to misdiagnosis as a result of metastasis from a malignant solid tumor. Cytomorphology and flow cytology indicated important directions for the diagnosis of the disease in the early stage. Combined with pathological and other examinations to confirm the diagnosis of isolated MS, administration of chemotherapy regimen containing cytarabine could prolong disease-free survival and time before progress to AML.

## Author contributions

Lilian Li, Li Wang and Wendong Ju collected and analyzed data, Kunyi Deng and Wenpeng Ni wrote the manuscript. All authors read and approved the final manuscript.

**Data curation:** Kunyi Deng, Lilian Li, Yanhui Chen, Li Wang, Wendong Ju.

**Formal analysis:** Wenpeng Ni.

**Funding acquisition:** Wenpeng Ni.

**Investigation:** Kunyi Deng, Lilian Li, Yanhui Chen, Wendong Ju.

**Resources:** Kunyi Deng, Li Wang.

**Supervision:** Kunyi Deng, Wenpeng Ni.

**Validation:** Wenpeng Ni.

**Writing – original draft:** Kunyi Deng, Wenpeng Ni.

**Writing – review & editing:** Kunyi Deng, Wenpeng Ni.

## Supplementary Material


