# Supplementary material for: Isolated myeloid sarcoma with pericardial and pleural effusions as first manifestation: A case report
Source: Medicine (Baltimore). 2022 Oct 21;101(42):e31026. doi: 10.1097/MD.0000000000031026 (PMC9592339; doi:10.1097/MD.0000000000031026)
Supplement: Supplementary file 1 [file medi-101-e31026-s001.pdf]

Supplementary figure 1 Images from the pleural effusion flow cytometry analysis. The results showed that about 66.1% of myeloid blasts were seen in pleural effusion specimens. Lymphocytes account for 21.5%, granulocytes account for 9.9%, monocytes account for 1.6%, CD45^{dim} cells account for 66.9%, and CD45^{-} cells account for 0.1%.

Markers Run: CD5、CD7、CD56、CD8、CD4、CD3、CD2、CD10、CD19、CD20、CD14、CD13、CD64、CD16、CD11b、CD15、CD36、CD33、CD34、CD117、CD71、HLADR、CD38、CD138、CD200、CD61、7AAD、CD45、sIg-Kappa、sIg-Lambda.

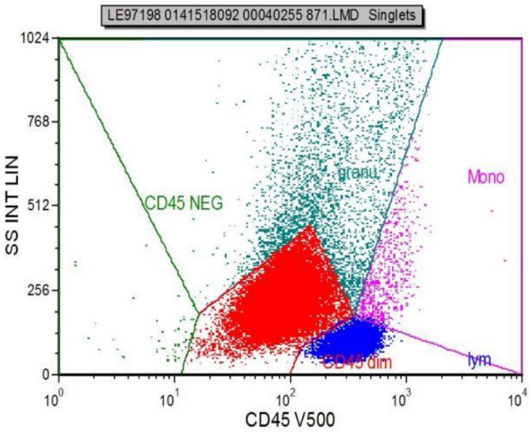

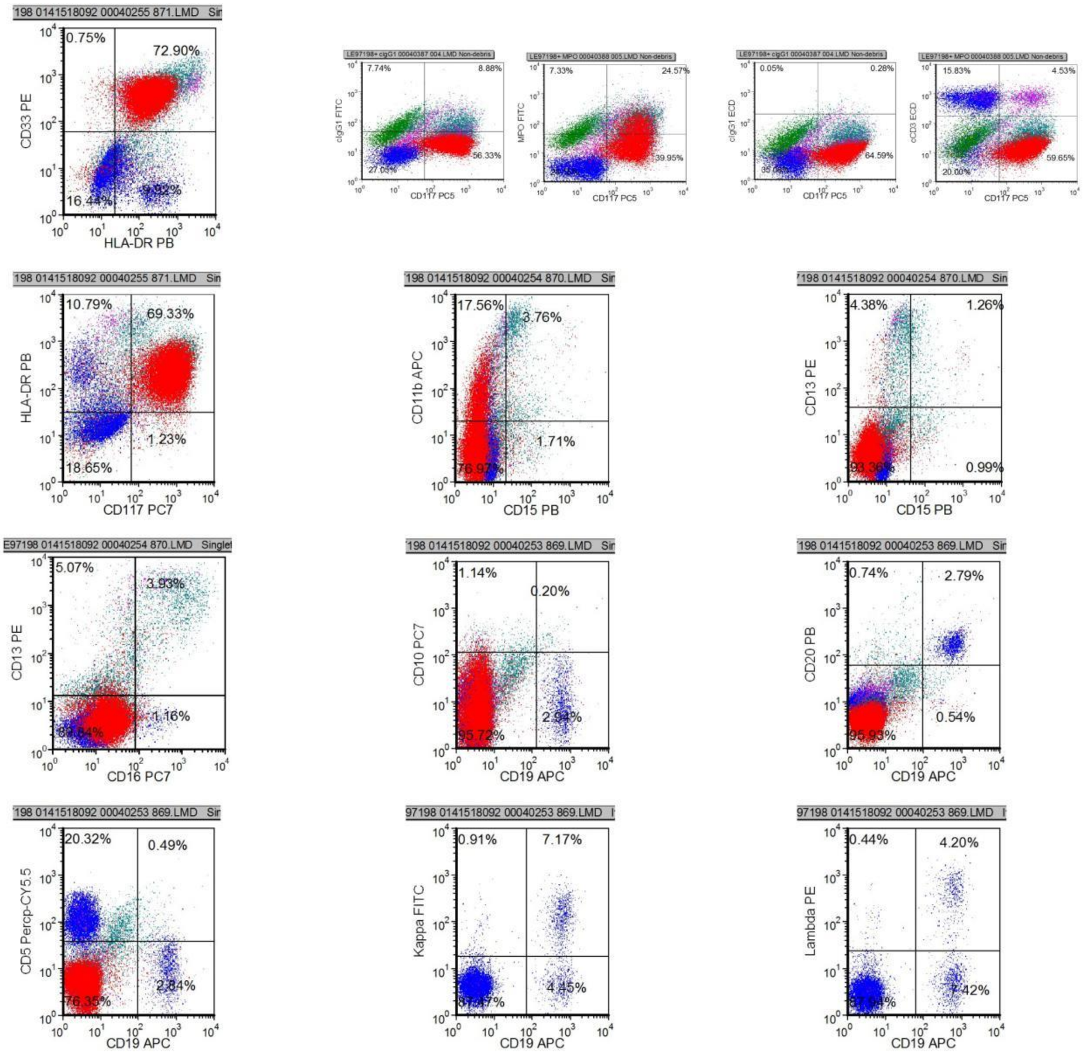
198 0141518092 00040255 871.LMD
CD33 PE
HLA-DR PB
0.75%
72.90%
16.44%
HLA-DR PB
CD117 PC7
10.79%
69.33%
1.23%
18.65%
198 0141518092 00040254 870.LMD
CD11b APC
CD15 PB
17.56%
3.76%
1.71%
CD13 PE
CD15 PB
4.38%
1.26%
0.99%
E97198 0141518092 00040254 870.LMD
CD13 PE
CD16 PC7
5.07%
3.93%
1.16%
198 0141518092 00040253 869.LMD
CD10 PC7
CD19 APC
1.14%
0.20%
95.72%
CD20 PB
CD19 APC
0.74%
2.79%
0.54%
95.93%
CD5 Percp-CY5.5
CD19 APC
20.32%
0.49%
76.35%
2.84%
Kappa FITC
CD19 APC
0.91%
7.17%
4.45%
Lambda PE
CD19 APC
0.44%
4.20%
7.42%
cIgG1 FITC
CD117 PC5
7.74%
8.88%
56.33%
MPO FITC
CD117 PC5
7.33%
24.57%
39.95%
cIgG1 ECD
CD117 PC5
0.05%
0.28%
64.59%
cCD3 ECD
CD117 PC5
15.83%
4.53%
20.00%
59.65%

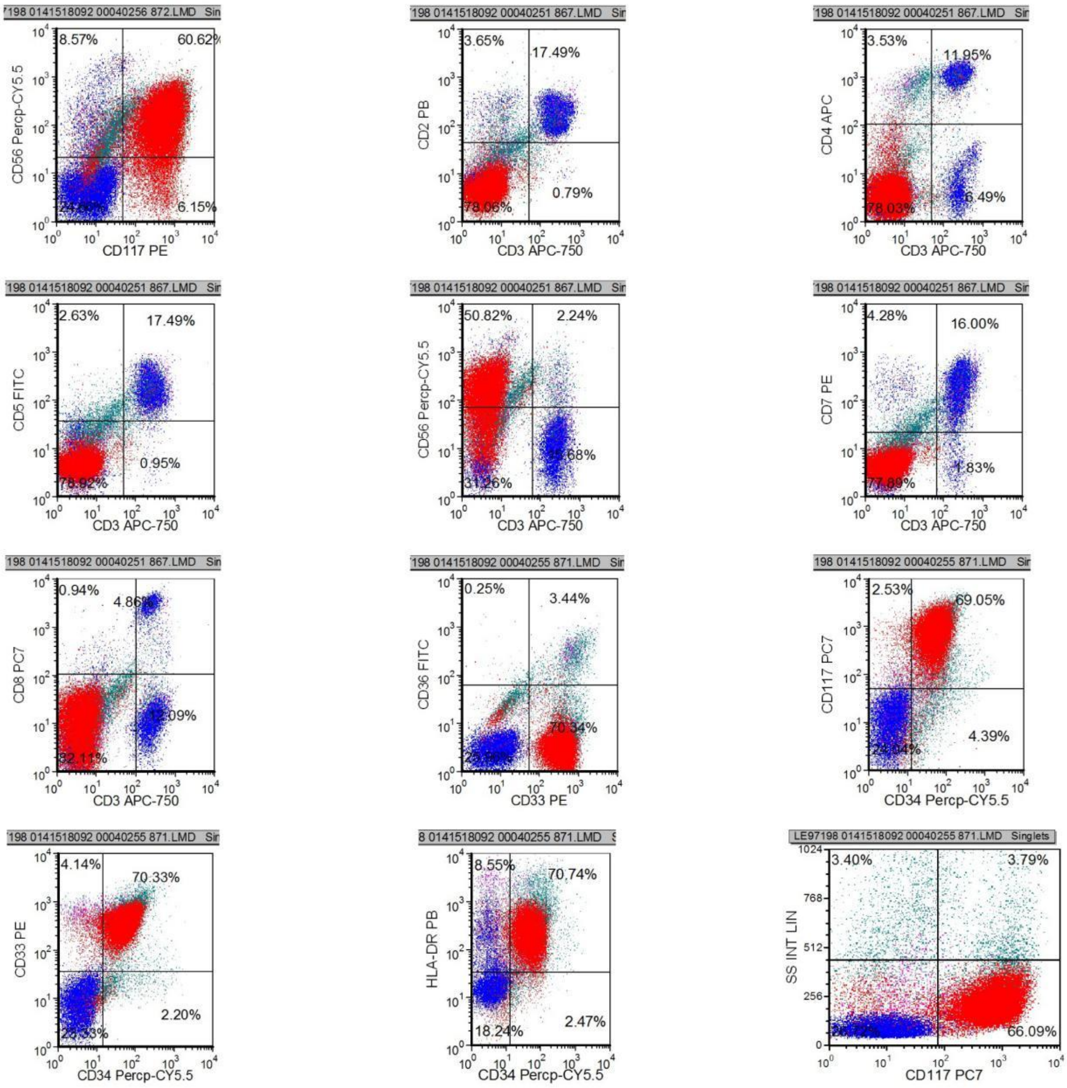
CD56 Percp-CY5.5
CD117 PE
8.57%
60.62%
6.15%
CD2 PB
CD3 APC-750
3.65%
17.49%
78.06%
0.79%
CD4 APC
CD3 APC-750
3.53%
11.95%
78.03%
6.49%
CD5 FITC
CD3 APC-750
2.63%
17.49%
0.95%
CD56 Percp-CY5.5
CD3 APC-750
50.82%
2.24%
31.26%
CD7 PE
CD3 APC-750
4.28%
16.00%
1.83%
CD8 PC7
CD3 APC-750
0.94%
CD36 FITC
CD33 PE
0.25%
3.44%
CD117 PC7
CD34 Percp-CY5.5
2.53%
69.05%
4.39%
CD33 PE
CD34 Percp-CY5.5
4.14%
70.33%
2.20%
HLA-DR PB
CD34 Percp-CY5.5
8.55%
70.74%
18.24%
2.47%
SS INT LIN
CD117 PC7
3.40%
3.79%
66.09%

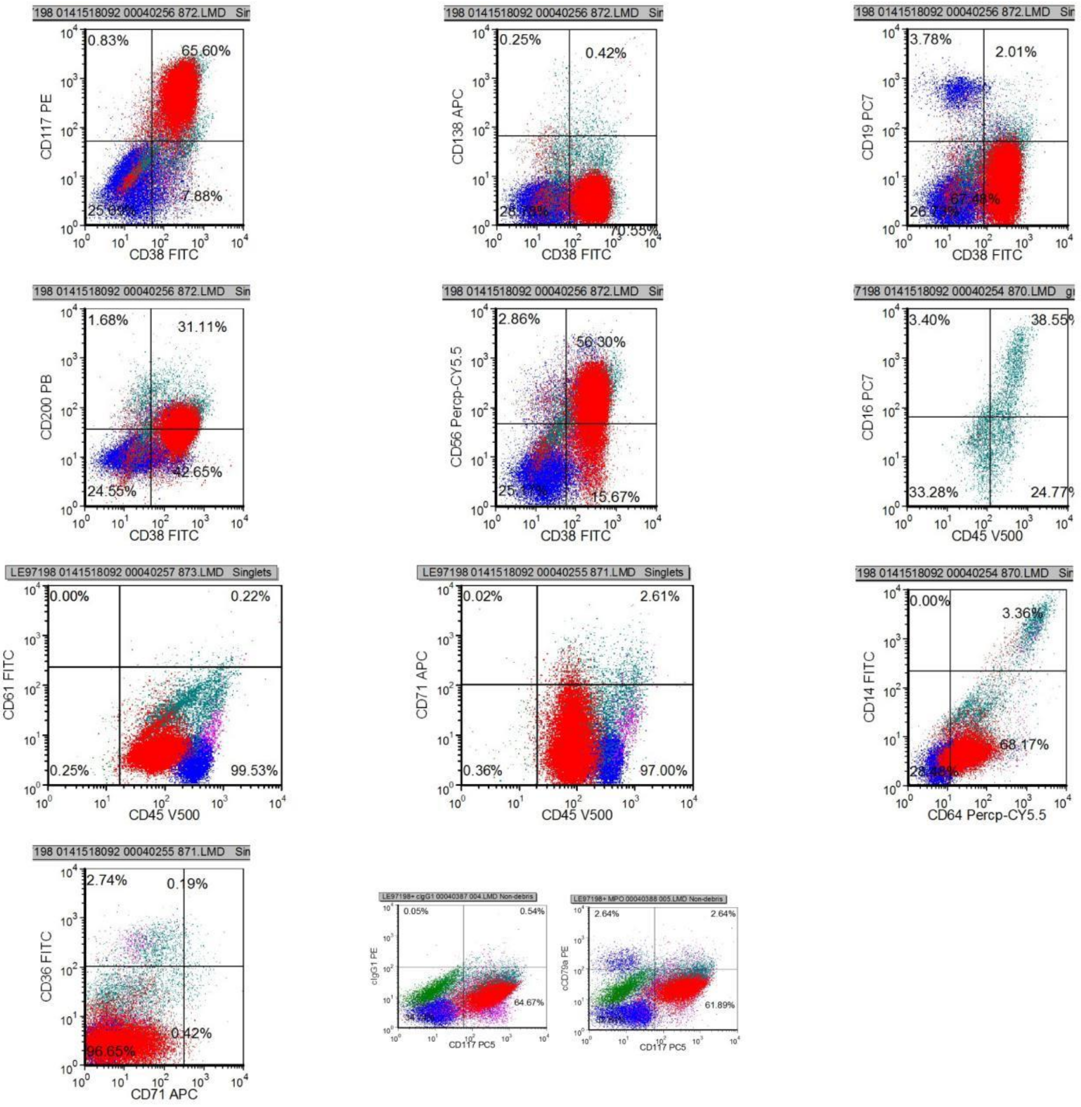
198 0141518092 00040256 872.LMD Sin
CD117 PE
CD38 FITC
0.83%
65.60%
25.0?%
7.88%
CD138 APC
0.25%
0.42%
70.55%
CD19 PC7
3.78%
2.01%
67.48%
CD200 PB
1.68%
31.11%
24.55%
42.65%
CD56 Percp-CY5.5
2.86%
56.30%
15.67%
7198 0141518092 00040254 870.LMD
CD16 PC7
CD45 V500
3.40%
38.55%
33.28%
24.77%
LE97198 0141518092 00040257 873.LMD Singlets
CD61 FITC
0.00%
0.22%
0.25%
99.53%
LE97198 0141518092 00040255 871.LMD Singlets
CD71 APC
0.02%
2.61%
0.36%
97.00%
198 0141518092 00040254 870.LMD Sin
CD14 FITC
CD64 Percp-CY5.5
0.00%
3.36%
68.17%
198 0141518092 00040255 871.LMD Sin
CD36 FITC
2.74%
0.19%
96.65%
0.42%
cIgG1 PE
CD117 PC5
0.05%
0.54%
64.67%
cCD79a PE
2.64%
2.64%
61.89%
