# Supplementary material for: Isolated myeloid sarcoma with pericardial and pleural effusions as first manifestation: A case report
Source: Medicine (Baltimore). 2022 Oct 21;101(42):e31026. doi: 10.1097/MD.0000000000031026 (PMC9592339; doi:10.1097/MD.0000000000031026)
Supplement: Supplementary file 2 [file medi-101-e31026-s002.pdf]

Supplementary figure 2

Images from the bone marrow flow cytometry analysis. No myeloid blasts with abnormal immunophenotype were detected by flow cytometry.

Lymphocytes account for 13.2%, granulocytes account for 75.0%, monocytes account for 3.1%, $CD45^{dim}$ cells account for 0.8%, and $CD45^{-}$ cells account for 6.8%.

Markers Run: CD5、CD7、CD56、CD8、CD4、CD3、CD2、CD10、CD19、CD20、CD14、CD13、CD64、CD16、CD11b、CD15、CD36、CD33、CD34、CD117、CD71、HLADR、CD38、CD138、CD200、CD61、7AAD、CD45、sIg-Kappa、sIg-Lambda.

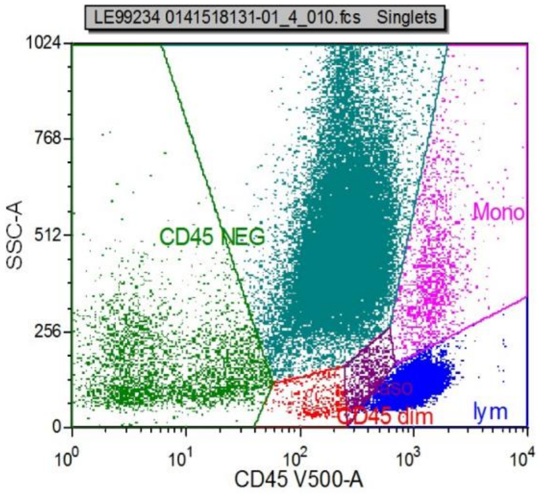

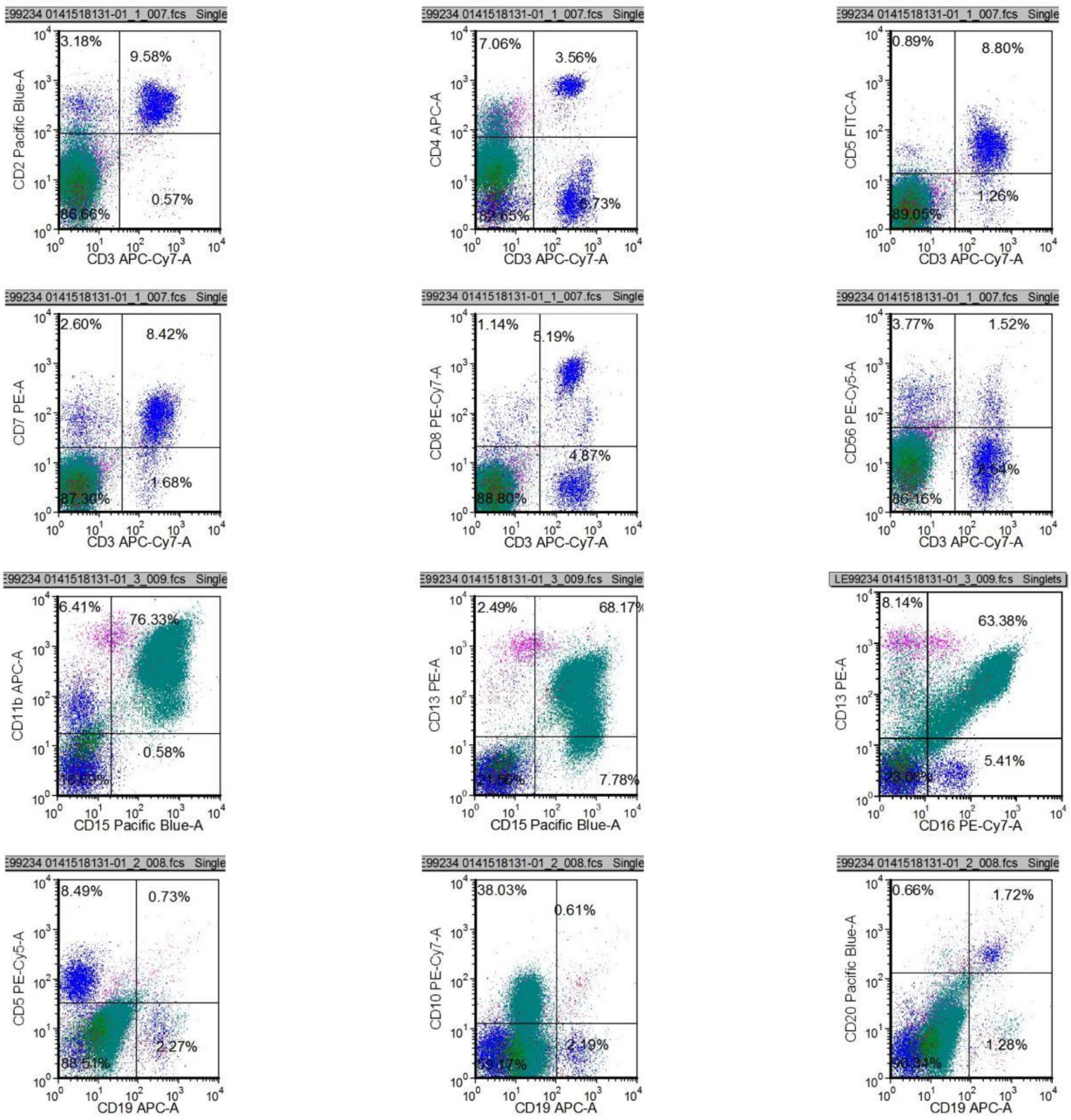

E99234 0141518131-01_1_007.fcs Single
CD2 Pacific Blue-A
CD3 APC-Cy7-A
3.18%
9.58%
0.57%
86.66%
CD4 APC-A
7.06%
3.56%
6.73%
82.65%
CD5 FITC-A
0.89%
8.80%
1.26%
89.05%
CD7 PE-A
2.60%
8.42%
1.68%
87.30%
CD8 PE-Cy7-A
1.14%
5.19%
4.87%
88.80%
CD56 PE-Cy5-A
3.77%
1.52%
86.16%
E99234 0141518131-01_3_009.fcs Single
CD11b APC-A
CD15 Pacific Blue-A
6.41%
76.33%
0.58%
CD13 PE-A
2.49%
68.17%
7.78%
LE99234 0141518131-01_3_009.fcs Singlets
CD16 PE-Cy7-A
8.14%
63.38%
5.41%
E99234 0141518131-01_2_008.fcs Single
CD5 PE-Cy5-A
CD19 APC-A
8.49%
0.73%
2.27%
CD10 PE-Cy7-A
38.03%
0.61%
2.19%
CD20 Pacific Blue-A
0.66%
1.72%
1.28%

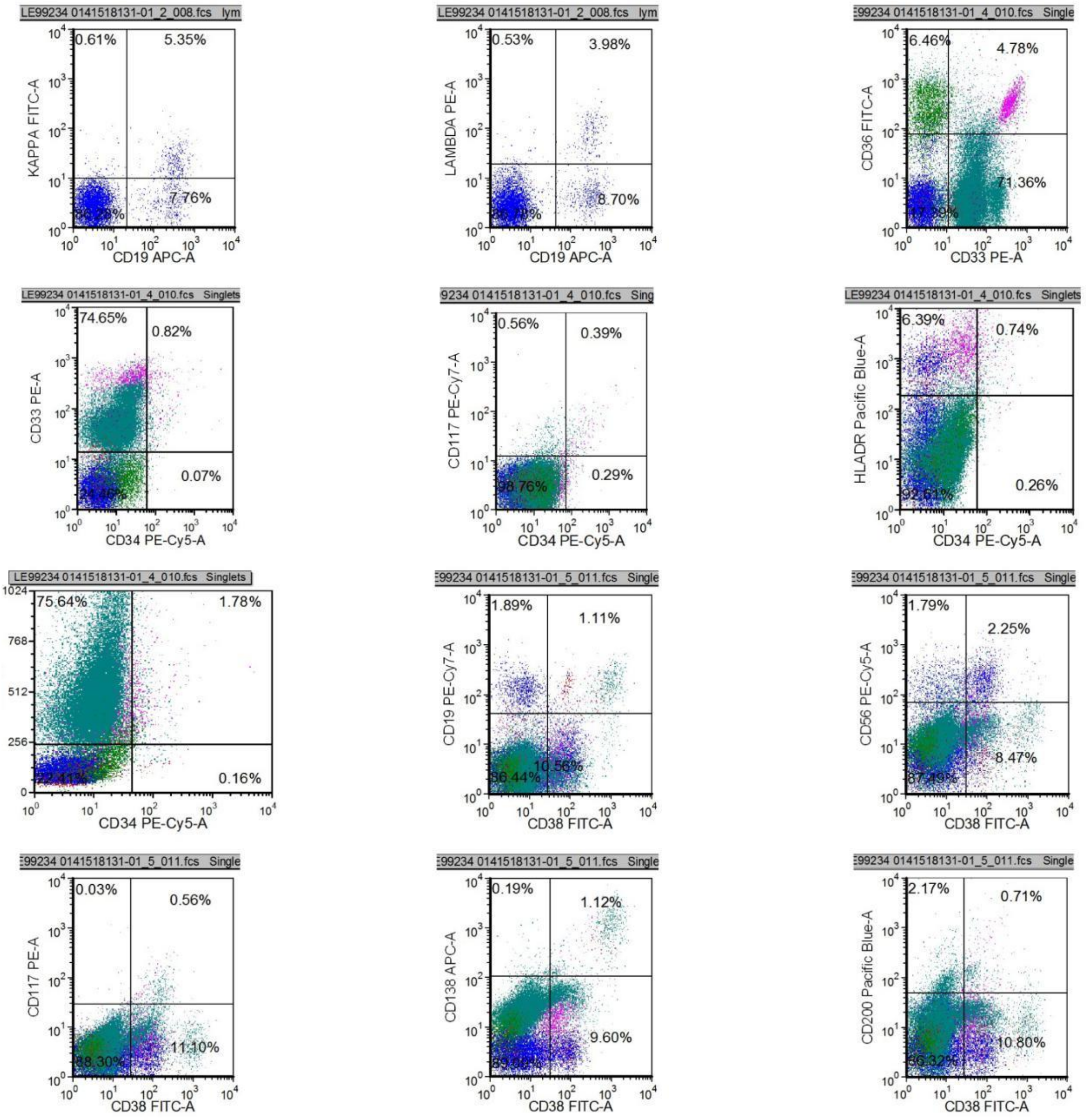

LE99234 0141518131-01_2_008.fcs lym
KAPPA FITC-A
CD19 APC-A
0.61%
5.35%
7.76%
LAMBDA PE-A
0.53%
3.98%
8.70%
E99234 0141518131-01_4_010.fcs Single
CD36 FITC-A
CD33 PE-A
6.46%
4.78%
71.36%
LE99234 0141518131-01_4_010.fcs Singlets
CD33 PE-A
CD34 PE-Cy5-A
74.65%
0.82%
0.07%
9234 0141518131-01_4_010.fcs Sing
CD117 PE-Cy7-A
0.56%
0.39%
98.76%
0.29%
HLADR Pacific Blue-A
6.39%
0.74%
92.61%
0.26%
75.64%
1.78%
0.16%
E99234 0141518131-01_5_011.fcs Single
CD19 PE-Cy7-A
CD38 FITC-A
1.89%
1.11%
10.56%
86.44%
CD56 PE-Cy5-A
1.79%
2.25%
8.47%
87.49%
CD117 PE-A
0.03%
0.56%
88.30%
11.10%
CD138 APC-A
0.19%
1.12%
9.60%
CD200 Pacific Blue-A
2.17%
0.71%
10.80%

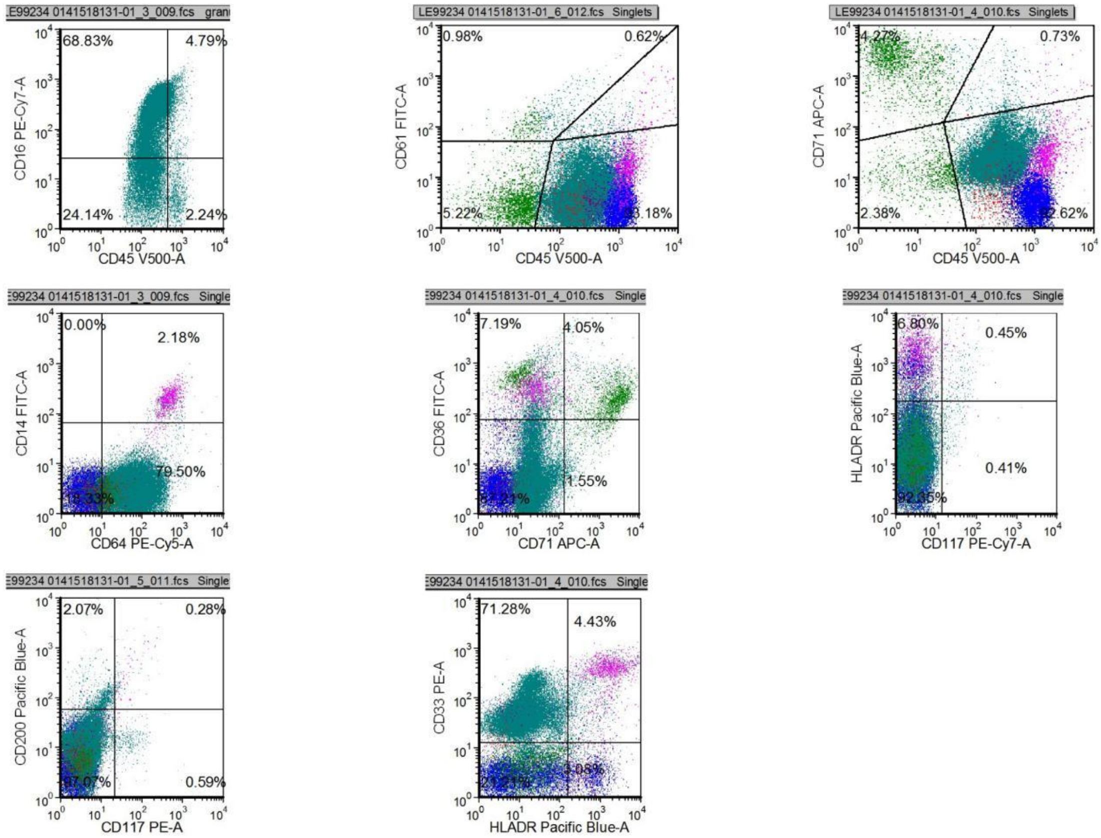

LE99234 0141518131-01_3_009.fcs
68.83%
4.79%
24.14%
2.24%
CD16 PE-Cy7-A
CD45 V500-A
LE99234 0141518131-01_6_012.fcs Singlets
0.98%
0.62%
5.22%
CD61 FITC-A
CD45 V500-A
LE99234 0141518131-01_4_010.fcs Singlets
0.73%
2.38%
CD71 APC-A
CD45 V500-A
0.00%
2.18%
79.50%
CD14 FITC-A
CD64 PE-Cy5-A
7.19%
4.05%
1.55%
CD36 FITC-A
CD71 APC-A
6.80%
0.45%
0.41%
HLADR Pacific Blue-A
CD117 PE-Cy7-A
2.07%
0.28%
0.59%
CD200 Pacific Blue-A
CD117 PE-A
71.28%
4.43%
CD33 PE-A
HLADR Pacific Blue-A
