# Supplementary material for: Isolated myeloid sarcoma with pericardial and pleural effusions as first manifestation: A case report
Source: Medicine (Baltimore). 2022 Oct 21;101(42):e31026. doi: 10.1097/MD.0000000000031026 (PMC9592339; doi:10.1097/MD.0000000000031026)
Supplement: Supplementary file 3 [file medi-101-e31026-s003.pdf]

Supplementary figure 3 Images from the bone marrow flow cytometry analysis. The results suggested that AML (non-M3).

Lymphocytes account for 6.0%, granulocytes account for 8.6%, monocytes account for 1.0%, $CD45^{dim}$ cells account for 81.8%, and $CD45^{-}$ cells account for 2.6%.

Markers Run: CD5、CD7、CD56、CD8、CD4、CD3、CD2、CD10、CD19、CD20、CD14、CD13、CD64、CD16、CD11b、CD15、CD36、CD33、CD34、CD117、CD71、HLADR、CD38、CD138、CD200、CD61、7AAD、CD45、sIg-Kappa、sIg-Lambda

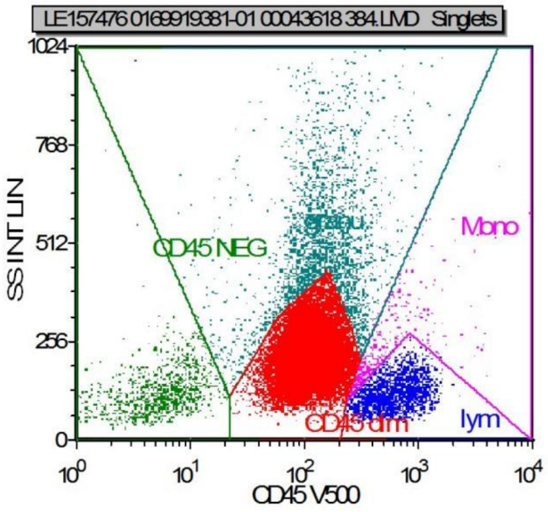

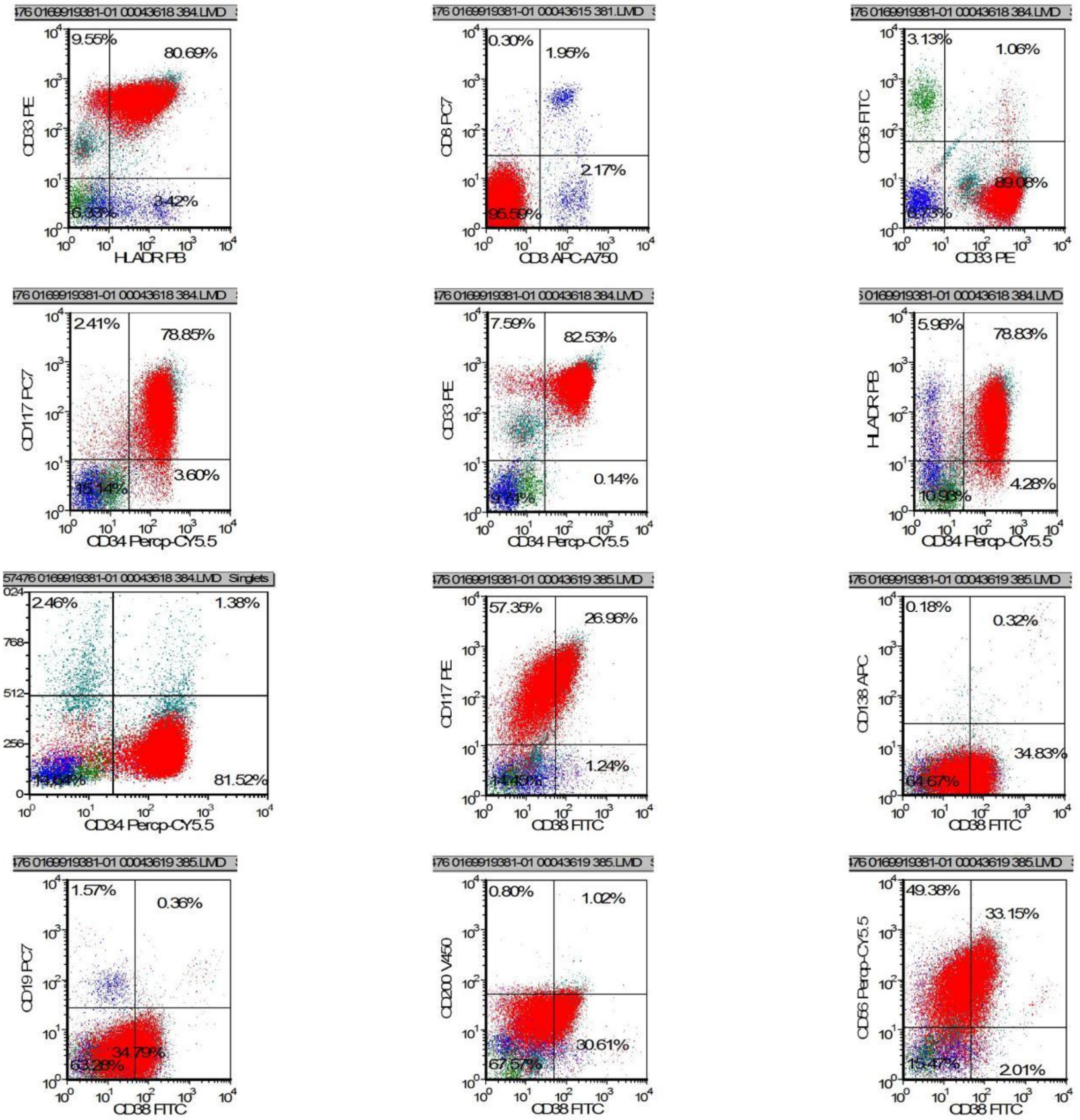

9.55%
80.69%
CD33 PE
HLADR PB
6.35%
0.30%
1.95%
CD8 PC7
CD3 APC-A750
2.17%
95.59%
3.13%
1.06%
CD36 FITC
CD33 PE
89.08%
2.41%
78.85%
CD117 PC7
CD34 Percp-CY5.5
15.14%
3.60%
7.59%
82.53%
CD33 PE
CD34 Percp-CY5.5
0.14%
5.96%
78.83%
HLADR PB
CD34 Percp-CY5.5
10.93%
4.28%
2.46%
1.38%
CD34 Percp-CY5.5
81.52%
57.35%
26.96%
CD117 PE
CD38 FITC
1.24%
0.18%
0.32%
CD138 APC
CD38 FITC
64.67%
34.83%
1.57%
0.36%
CD19 PC7
CD38 FITC
63.28%
34.79%
0.80%
1.02%
CD200 V450
CD38 FITC
67.57%
30.61%
49.38%
33.15%
CD56 Percp-CY5.5
CD38 FITC
15.47%
2.01%

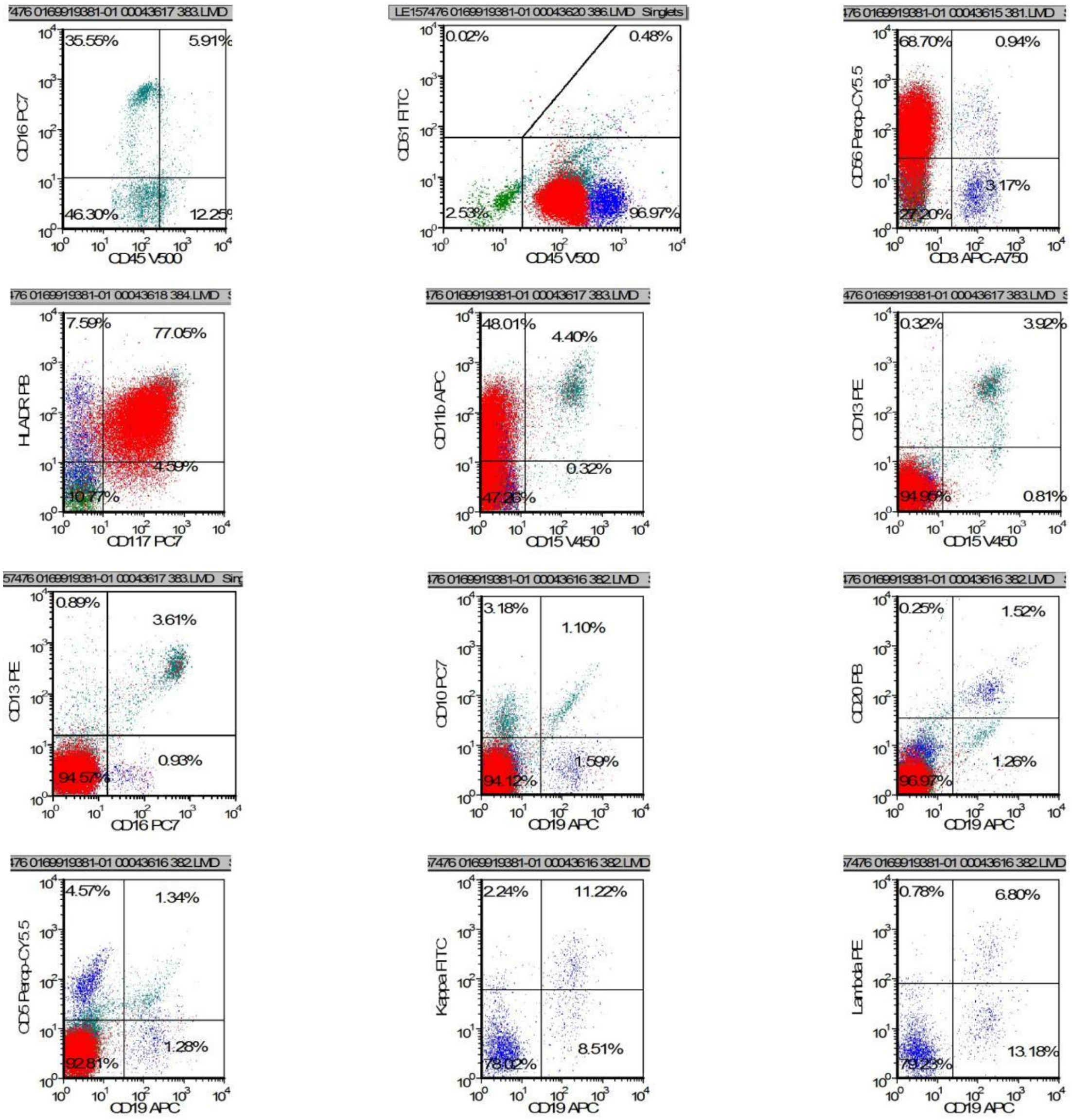

7476 0169919381-01 00043617 383.LMD
CD16 PC7
CD45 V500
35.55%
5.91%
46.30%
12.25%
LE157476 0169919381-01 00043620 386.LMD Singlets
CD61 FITC
CD45 V500
0.02%
0.48%
2.53%
96.97%
476 0169919381-01 00043615 381.LMD
CD56 Percp-CY5.5
CD3 APC-A750
68.70%
0.94%
27.20%
3.17%
476 0169919381-01 00043618 384.LMD
HLADR PB
CD117 PC7
7.59%
77.05%
10.77%
4.59%
476 0169919381-01 00043617 383.LMD
CD11b APC
CD15 V450
48.01%
4.40%
47.26%
0.32%
476 0169919381-01 00043617 383.LMD
CD13 PE
CD15 V450
0.32%
3.92%
94.95%
0.81%
57476 0169919381-01 00043617 383.LMD
CD13 PE
CD16 PC7
0.89%
3.61%
94.57%
0.93%
476 0169919381-01 00043616 382.LMD
CD10 PC7
CD19 APC
3.18%
1.10%
94.12%
1.59%
476 0169919381-01 00043616 382.LMD
CD20 PB
CD19 APC
0.25%
1.52%
96.97%
1.26%
476 0169919381-01 00043616 382.LMD
CD5 Percp-CY5.5
CD19 APC
4.57%
1.34%
92.81%
1.28%
57476 0169919381-01 00043616 382.LMD
Kappa FITC
CD19 APC
2.24%
11.22%
78.02%
8.51%
57476 0169919381-01 00043616 382.LMD
Lambda PE
CD19 APC
0.78%
6.80%
79.25%
13.18%

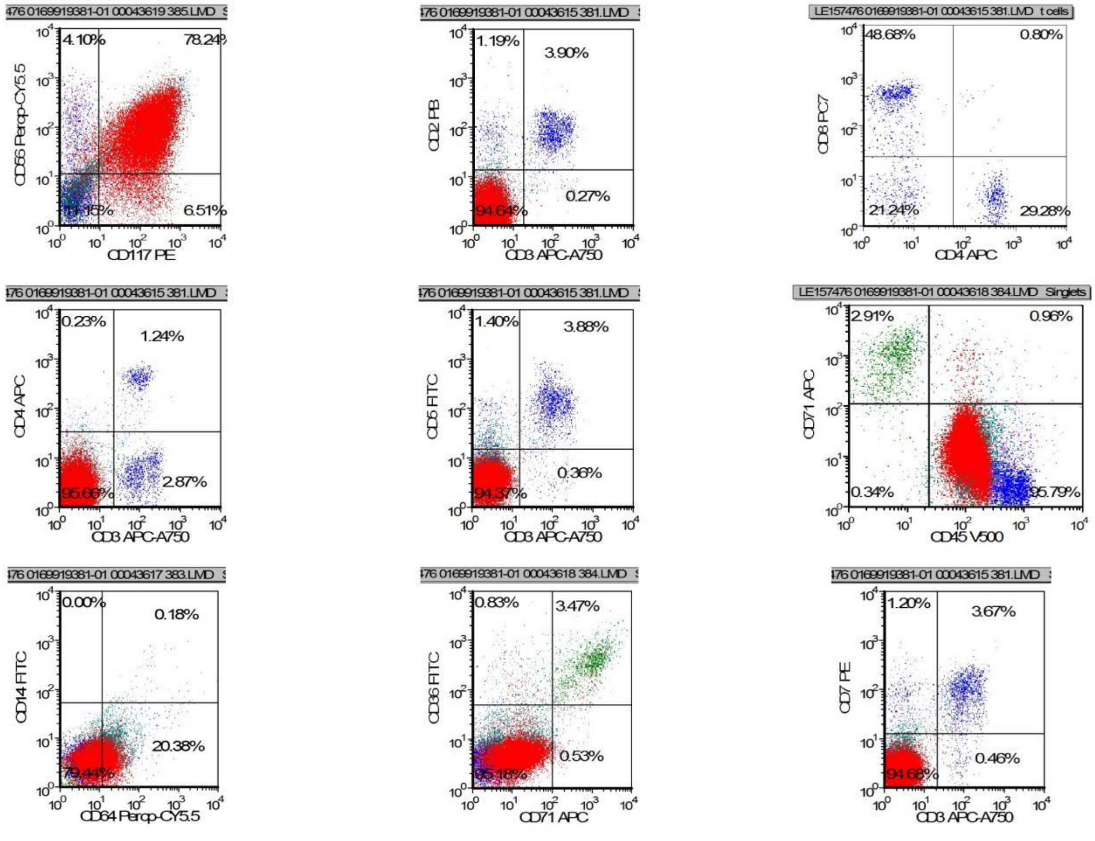

CD56 Percp-CY5.5
CD117 PE
4.10%
78.24%
6.51%
CD2 PB
CD3 APC-A750
1.19%
3.90%
0.27%
94.64%
LE157476 0169919381-01 00043615 381.LMD t cells
CD8 PC7
CD4 APC
48.68%
0.80%
21.24%
29.28%
CD4 APC
CD3 APC-A750
0.23%
1.24%
2.87%
CD5 FITC
CD3 APC-A750
1.40%
3.88%
0.36%
94.37%
LE157476 0169919381-01 00043618 384.LMD Singlets
CD71 APC
CD45 V500
2.91%
0.96%
0.34%
CD14 FITC
CD64 Percp-CY5.5
0.00%
0.18%
20.38%
79.44%
CD36 FITC
CD71 APC
0.83%
3.47%
0.53%
CD7 PE
CD3 APC-A750
1.20%
3.67%
0.46%
94.68%
